# Development of a two-step protocol for culture expansion of human annulus fibrosus cells with TGF-β1 and FGF-2

**DOI:** 10.1186/s13287-016-0332-1

**Published:** 2016-07-12

**Authors:** Po-Hsin Chou, Shih-Tien Wang, Hsiao-Li Ma, Chien-Lin Liu, Ming-Chau Chang, Oscar Kuang-Sheng Lee

**Affiliations:** Department of Orthopedics and Traumatology, Taipei Veterans General Hospital, Taipei city, Taiwan; School of Medicine, National Yang-Ming University, Taipei city, Taiwan; Institute of Clinical Medicine, National Yang-Ming University , Taipei city, Taiwan; Department of Medical Research, Taipei Veterans General Hospital, Taipei city, Taiwan; Taipei City General Hospital, No.145, Zhengzhou Rd., Datong Dist., Taipei City, 10341 Taiwan (R.O.C.)

**Keywords:** Disc degeneration, Annulus fibrosus, Mitogen, Morphogen, FGF-2, TGF-β1, Tissue engineering

## Abstract

**Background:**

Different biologic approaches to treat disc regeneration, including growth factors (GFs) application, are currently under investigation. Human annulus fibrosus (hAF) repair or regeneration is one of the key elements for maintenance and restoration of nucleus pulposus function. However, so far there is no effective treatment for this purpose. The aim of the present study was to investigate the response of hAF cells to different combinations of GFs, and develop a protocol for efficient culture expansion.

**Methods:**

hAF cells were harvested from degenerated disc tissues during surgical intervertebral disc removal, and hAF cells were expanded in a monolayer. The experiments were categorized based on different protocols with transforming growth factor (TGF-β1) and fibroblast growth factor (FGF-2) culture for 14 days: group 1 had no GFs (control group); group 2 received TGF-β1; group 3 received FGF-2; group 4 received both GFs; and group 5 (two-step) received both GFs for the first 10 days and TGF-β1 only for the next 4 days. Cell proliferation, collagen, and noncollagen extracellular matrix (ECM) production and genes expression were compared among these groups.

**Results:**

At days 3, 7 and 10 of cultivation, groups 4 and 5 had significantly more cell numbers and faster cell proliferation rates than groups 1, 2, and 3. At 14 days of cultivation, significantly more cell numbers were observed in groups 3 and 4 than in group 5. The group 4 had the most cell numbers and the fastest proliferation rate at 14 days of cultivation. After normalization for cell numbers, group 5 (two-step) produced the most collagen and noncollagen ECM at 10 and 14 days of cultivation among the five groups. In group 5, ECM gene expression was significantly upregulated. High expression of matrix metalloproteinase-1 was upregulated with FGF-2 on the different days as compared to the other groups. Annulus fibrosus cell phenotypes were only marginally retained under the different protocols based on quantitative polymerase chain reaction results.

**Conclusion:**

Taken together, the two-step protocol was the most efficient among these different protocols with the most abundant ECM production after normalization for cell numbers for culture expansion of hAF cells. The protocol may be useful in further cell therapy and tissue engineering approaches for disc regeneration.

## Background

Back pain resulting from degenerative disc disease (DDD) is one of the most common causes of disability worldwide [1]. The process of intervertebral disc degeneration is an aberrant, cell-mediated response to progressive structural failures which is evident on routine clinical imaging studies [2]. Moreover, the pathogenesis of DDD is still a complicated process [2] with tissue weakening resulting from genetic inheritance, aging, nutritional compromise, and mechanical loading [1].

Previous studies have indicated a strong association between human annulus fibrosus (hAF) tear (circumferential tear, radial fissure, or peripheral rim) and disc degeneration [3]. As an integral part of the disc, the hAF plays an essential role in the pathogenesis of disc degeneration. It has been reported that the hAF sealing augmentation method and regeneration could facilitate nucleus pulposus (NP) tissue engineering [4]. Disc degeneration is known to accompany hAF biochemical changes, and hAF might still play an important role in disc degeneration [3].

Major components of the extracellular matrix (ECM) in hAF are water, collagens (60–70 % dry weight; mostly type I and less so type III [1, 5]), and proteoglycan (15–20 % dry weight) [6]. The hAF cells regulate ECM homeostasis with a variety of stimuli, including cytokines and growth factors (GFs), and maintain their steady state in the normal disc [7, 8]. Disc degeneration may result from an imbalance between anabolic and catabolic processes and loss of the steady metabolism [5]. Evidence has indicated that matrix metalloproteinases (for example, MMP-1 and MMP-3) and aggrecanases (ADAMTS-4) were upregulated [6, 9], and has revealed a catabolic state and possible pathogenic roles in ECM degradation that are characterized in DDD, including hAF [6].

GFs in vitro acted as anabolic regulators of hAF cell metabolism; the GFs included insulin-like growth factor (IGF)-1 [10–12], transforming growth factor (TGF)-β1 [10, 13], fibroblast growth factor (FGF)-2 [12], and platelet-derived growth factor (PDGF) [11, 12]. These studies indicated that different GFs had different anabolic effects on hAF cells in either a monolayer [11, 12] or three-dimensional cultures in agarose or alginate [10, 13].

FGF-2 [14] was described as a mitogen for fibroblastic cells and as being involved in tissue regeneration. Regarding morphogen, TGF-β1 was superior to IGF-1, PDGF, and FGF-2 in upregulating aggrecan synthesis [15, 16]. Increased expression of FGF-2 and TGF-β1 and its receptors were found in human degenerated or herniated discs [17, 18]. It was assumed that disc degeneration stimulated or triggered the release of FGF-2 and TGF-β1 by the disc itself. Consequently, these two GFs may play important roles in biologic repair or rescue of the degeneration. With tissue engineering for hAF cells, one of the key elements is to amplify large numbers of cells and produce more ECM [4] simultaneously. Studies [10–13] have evaluated the effects of continuous isolated single or combined GF treatment for tissue expansion of hAF cells in vitro. However, no study has compared the results regarding culture expansion of hAF cells under different combination of GFs.

The objective of this study was to evaluate cell proliferation and ECM production of hAF cells harvested from degenerated disc tissues under different combinations of GFs, consisting of FGF-2 and TGF-β1 in vitro. We hypothesize that hAF cells harvested from degenerated disc tissues respond differently to combinations of GFs.

## Methods

The study was approved by the ethics committee of Taipei Veterans General Hospital. Human disc tissues were obtained from six patients (four female and two male donors; mean age 57 years, range 52–61 years) diagnosed as having lumbar spondylolisthesis, and who underwent transforaminal interbody fusion (TLIF) surgery. The patients signed informed consent to participate before surgery.

To exclude contamination by the inner NP tissues, the most inner AF layers were discarded. hAF tissue from degenerated disc tissues were finely minced prior to enzymatic digestion. hAF tissues were digested for 30 min at 37 °C under gentle agitation in phosphate-buffered saline (PBS) containing 0.1 % collagenase, and then digested at room temperature for another 30 to 40 min with gentle sterile agitation under a laminar flow hood.

After the hAF tissues had been digested for 1 h, the tiny fragmented tissues were passed through filters, centrifuged, and cultured in dishes that were supplemented with Dulbecco’s modified Eagle medium (DMEM; Sigma-Aldrich, St. Louis, MO, USA) with 10 % fetal bovine serum (FBS; HyClone, Logan, UT, USA), 100 UI penicillin, 1000 UI streptomycin, and 2 mm l-glutamine (Invitrogen, Carlsbad, CA, USA). The dishes were incubated at 37 °C in a humidified atmosphere of 5 % CO_2_, 95 % air under normoxia (20 % O_2_). A change of medium was carried out twice a week.

Once hAF cells (passage 0) reached 70–80 % confluence, cells were detached with 0.25 % trypsin–ethylenediaminetetraacetic acid (Gibco BRL), washed twice with PBS, centrifuged at 1000 rpm for 4 min, and cultured in the P-100 flask (passage 1). After reaching 70–80 % confluence, the cells were harvested and expanded in the flask using the same methods (passage 2). Once passage 2 cells had reached confluence, the hAF cells were detached by trypsin, washed with PBS, and cryopreserved in liquid nitrogen in FBS with 10 % dimethyl sulfoxide (DMSO). Cells from passage 3 were used for the subsequent experiments to avoid dedifferentiation when cultured in vitro after a long passage (greater than passage 6) [19], which could interfere with the experimental results.

**Fig. 1:**
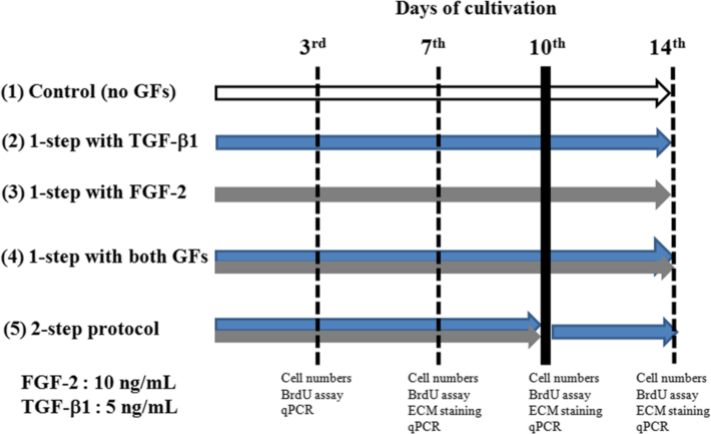
Experimental flowchart and the five groups using one-step and two-step protocols consisting of TGF-β1 and FGF-2 treatment for 14 days. Group 1: control group with no growth factor (*GF*). Group 2: one-step protocol with TGF-β1 5 ng/mL. Group 3: one-step protocol with FGF-2 10 ng/mL. Group 4: one-step protocol with treatment with both GFs. Group 5: two-step protocol with treatment with both GFs for the first 10 days and then TGF-β1 only for the next 4 days. *BrdU* 5-bromo-2’-deoxyuridine, *ECM* extracellular matrix, *FGF* fibroblast growth factor, *qPCR* quantitative polymerase chain reaction, *TGF* transforming growth factor

TGF-β1 and FGF-2 (Sigma-Aldrich) were prepared following the manufacturer’s instructions, and were added to the LG-DMEM medium to achieve a working concentration of 5 ng/mL TGF-β1 and 10 ng/mL FGF-2. The five groups were categorized using different protocols consisting of TGF-β1 and FGF-2 treatment for 14 days (Fig. 1): group 1 was a control group with no GFs; groups 2, 3, and 4 were one-step protocols with TGF-β1 or FGF-2 or combined GF treatment, respectively; group 5 was a two-step protocol, with both GFs for the first 10 days and then TGF-β1 treatment only for the next 4 days. FBS was not used in any of the experimental groups, including the control group. To generate starvation, cells were cultured in DMEM medium with Corning™ ITS (insulin, transferrin, selenium) solution for 24 h.

Aprroximately 3 × 10^4^ cells were placed in P60 dishes for the five groups. Cultured media were changed twice a week. Serial passage was performed when cells reached confluence, usually at days 7 and 10. Cell numbers were summed together during passage. Cell numbers were determined on days 3, 7, 10, and 14 by trypan blue exclusion in a Neubauer counting chamber. Three independent experiments were performed at each time point.

A total of 1.5 × 10^3^ cells were placed in each well of a 96-well plate. For cell proliferation, we used the Cell Proliferation ELISA BrdU assay (Roche Molecular Biochemicals, Mannheim, Germany), according to the manufacturer’s protocol, on days 3, 7, 10, and 14. Briefly, cells were labeled with 10 uM 5-bromo-2’-deoxyuridine (BrdU) for 1 h. The cells were then fixed, and DNA was denatured for 30 min. After 1 h of incubation with a peroxidase-coupled anti-BrdU antibody, cells were washed three times with PBS. Thereafter, peroxidase substrate (tetramethylbenzidine) was added for 30 min and chemiluminescence measurements were performed on a luminometer (Tecan’s Infinite M1000 microplate reader). Three independent experiments were performed at each time point.

Collagen and noncollagen ECM proteins were quantified by colorimetric analyses, as described previously [20, 21], on days 7, 10 and 14. Selective binding of Sirius Red F3BA to collagen and Fast Green FCF (Sigma Chemical) to noncollagen protein was used when both were dissolved in aqueous saturated picric acid. In all, 3 × 10^4^ cells in each well of a six-well plate were incubated with 1 mL saturated picric acid solution that contained 0.1 % Sirius Red F3BA and 0.1 % Fast Green FCF. The plates were incubated at room temperature for 30 min in a rotary shaker. The fluids were then carefully withdrawn, and the plates were washed two to three times with distilled water until the fluid was colorless. After washing with water, 1 mL 1:1 (equal volume) 0.1 % NaOH and absolute methanol was added to each well to elute the color. The elute color was immediately analyzed with a spectrophotometer at 540 and 605 nm (U3300 Pro; Amersham Biosciences, Freiburg, Germany), respectively. Three independent experiments were performed at each time point.

Cells (3 × 10^4^ seeded in P60 dishes for the five groups) were subcultured when confluence was reached. Targeted genes were analyzed at 3, 7, 10, and 14 days by quantitative real-time polymerase chain reaction (PCR). Total RNA was extracted using an RNeasy Mini Kit (QIAGEN, Stanford, Valencia, CA, USA). RNA samples were used for reverse transcription and subsequent PCR amplification. Quantitative real-time PCR was performed with a LightCycler 480 Real-Time System (Roche Applied Sciences, Mannheim, Germany). Intron spanning primers for targeted genes were designed by the Universal ProbeLibrary Assay Design Center and detected with corresponding probes (Roche) (Table 1). The glyceraldehyde-3-phosphate dehydrogenase (GAPDH) housekeeping gene was used for endogenous internal control. All targeted genes were normalized using GAPDH and analyzed using the 2^–△△Ct^ method expressed as fold-change. Three independent experiments were performed at each time point.

**Table 1:** Primers used in the real-time polymerase chain reaction analysis

Target gene	Forward primer	Reverse primer	GenBank accession no.	Roche probe no.
Housekeeping gene				
*GAPDH*	agccacatcgctcagacac	gcccaatacgaccaaatcc	NM_002046	#60
Anabolic genes				
*COL IA1*	gggattccctggacctaaag	ggaacacctcgctctcc	NM_000088	#67
*COL IIIA1*	ctggaccccagggtcttc	catctgatccagggtttcca	NM_000090	#20
*Aggrecan*	cctccccttcacgtgtaaaa	gctccgcttctgtagtctgc	NM_01135	#76
Catabolic genes				
*MMP-1*	gctaacctttgatgctataactacga	tttgtgcgcatgtagaatctg	NM_001145938.1	#7
*MMP-3*	caaaacatatttctttgtagaggacaa	ttcagctatttgcttgggaaa	NM_002422	#36
*ADAMTS-4*	ccaggcactgggctactact	gaacagggggtcccatcta	NM_005099	#67
AF putative genes				
*COL1A1*	gggattccctggacctaaag	ggaacacctcgctctcc	NM_000088	#67
*COL2A1*	tggtgctaatggcgagaag	cccagtctctccacgttcac	NM_033150.2	#4
*COL5A1*	gggattccctggacctaaag	ggaacacctcgctctcc	NM_000093.3	#42
*COL12A1*	cctggatgaggaggtgtttg	cagcactggcgacttagaaa	NM_004370.5	#89
*SFRP-2*	gctagcagcgaccacctc	tttttgcaggcttcacatacc	NM_003013.2	#83

*ADAMTS* a disintegrin and metallopeptidase with thrombospondin motif, *COL* collagen, *GAPDH* glyceraldehyde-3-phosphate dehydrogenase, *MMP* matrix metallopeptidase, *SFRP* secreted frizzled-related protein

For each disc sample, three replications were performed in each experiment for cell number, cell proliferation, picric acid stain, and qPCR analysis. Statistical significance among groups was analyzed using the repeated-measure analysis of variance (ANOVA) test with post hoc test (Bonferroni test) (SPSS 15.0; SPSS, Chicago, IL, USA). A *p* value less than 0.05 indicated statistical significance.

## Results

**Fig. 2:**
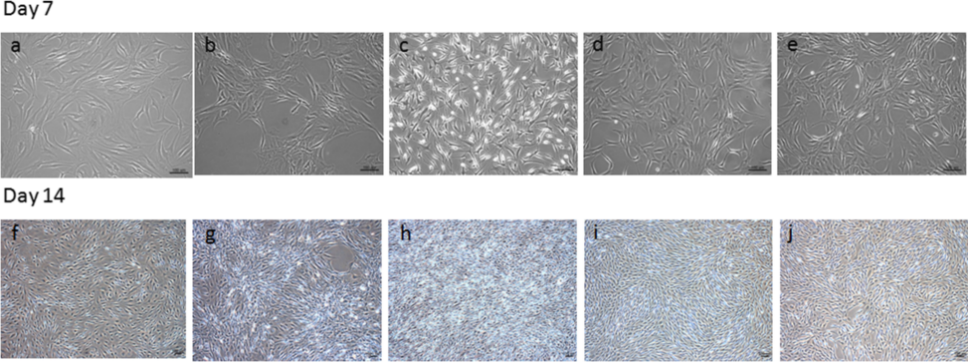
Morphology of hAF cells harvested from degenerated disc tissues after GF treatment in the five groups. Control group at **(a)** 7 days and **(f)** 14 days. TGF-β1 group at **(b)** 7 days and **(g)** 14 days. FGF-2 group at **(c)** 7 days and **(h)** 14 days. Treatment group with both GFs at **(d)** 7 days and **(i)** 14 days. Two-step group at **(e)** 7 days and **(j)** 14 days. *Scale bar* = 100 μm

The doubling time of hAF cells was approximately 67.8 ± 11 h in primary culture. hAF cells had astrocyte-like morphology with one or three protrusions in a primary monolayer. Cells treated without GFs had a morphology similar to those cultured in a monolayer (Fig. 2a, f). Cells in group 2 (TGF-β1) had a more flattened shape; these cells tended to aggregate to form linear or circular multiple cell complexes when cell contact occurred (Fig. 2b, g). Cells in group 3 (FGF-2) had more homogenous smaller cells, with short cell processes (Fig. 2c, h). Groups 4 (combined GFs; Fig. 2d, i) and 5 (two-step; Fig. 2e, j) had a mixed cellular morphology.

**Fig. 3:**
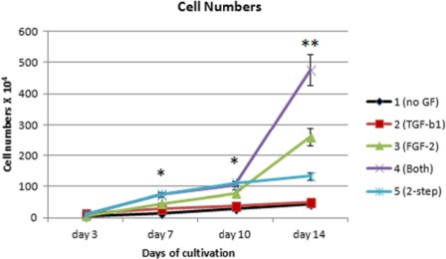
Cell numbers in the five groups. Approximately 3 × 10^4^ hAF cells were placed in each P60 dish and cultured. Cells were harvested and counted at days 3, 7, 10, and 14. The results were averaged and expressed as the mean ± standard deviation. Each point indicates the mean of three experiments in the same group (*n* = 6). *Cell numbers were significantly greater in groups 4 and 5 than in groups 1, 2, and 3 at 7 and 10 days of cultivation. **Cell numbers in groups 3 and 4 were significantly greater than in group 5 at 14 days of cultivation. *FGF* fibroblast growth factor, *GF* growth factor, *TGF* transforming growth factor

**Fig. 4:**
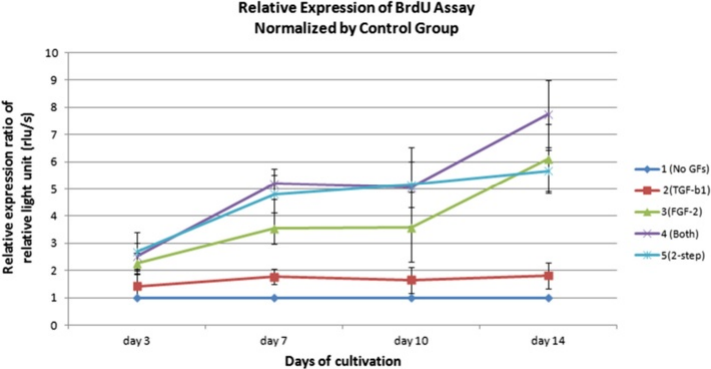
Relative expression of the BrdU results in the five groups normalized by the control group. hAF cells (1500) were placed in each well of a 96-well plate for the five groups. Cell proliferation was evaluated by luminometer. Each point indicates the mean of three experiments in the same group (*n* = 6). The results of the relative expression ratio in the different groups were normalized by the control group at different days of cultivation. The relative expression ratio was expressed as the mean ± standard deviation. At days 3, 7 and 10 of cultivation, groups 4 and 5 had significantly faster cell proliferation rates than groups 1, 2, and 3. The group 4 had the fastest cell proliferation rate at 14 days of cultivation. *BrdU* 5-bromo-2’-deoxyuridine, *FGF* fibroblast growth factor, *GF* growth factor, *TGF* transforming growth factor

At 7 and 10 days of cultivation, the cell numbers were significantly higher in groups 4 and 5 than in groups 1, 2, and 3. Up to day 10, both GFs were used in groups 4 and 5. These GFs might have a synergistic effect on cell growth. At 14 days of cultivation, the cell numbers in groups 3 and 4 were significantly 1.95 and 3.58 times higher, respectively, than those in group 5 (Fig. 3). At 3, 7 and 10 days of cultivation, groups 4 and 5 had significantly faster cell proliferation rates than groups 1, 2, and 3 after normalization by the control group. The group 4 had the fastest proliferation rate at 14 days of cultivation. The cell numbers results were compatible with the proliferation results. (Fig. 4).

**Fig. 5:**
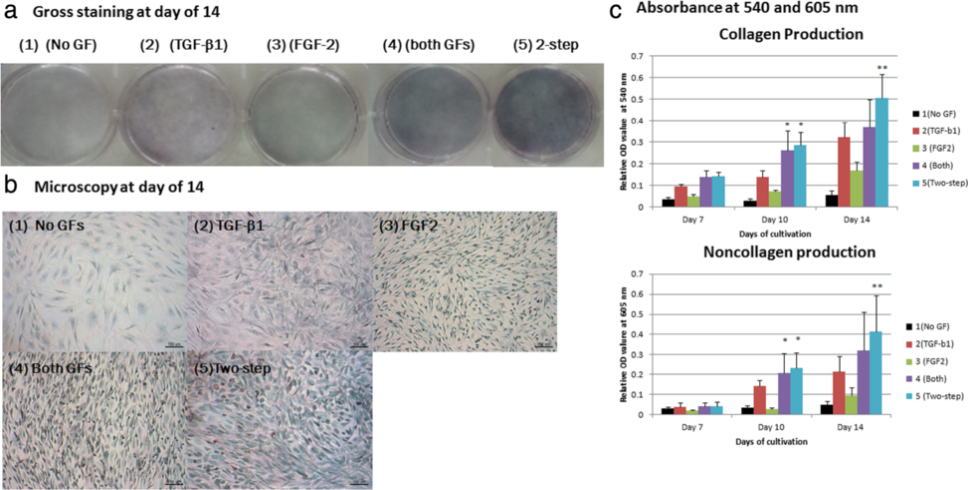
Collagen and noncollagen ECM protein production in the five groups. hAF cells (3 × 10^4^ cells) were placed in each well of a six-well plate for the five groups. The culture plates were stained with Sirius Red and Fast Green for collagen and noncollagen proteins at days 7, 10, and 14, eluted, and absorbance was evaluated with a spectrophotometer at 540 and 605 nm, respectively. Each bar indicates the mean of three experiments in the same group (*n* = 6). **a** Gross staining in the five groups at day 14. **b** Microscopic pictures were taken in the five groups at 14 days. *Scale bar*s = 100 μm. **c** Absorbance is shown (mean ± standard deviation). The value at each bar is the mean of three experiments in the same group. *Significantly more collagenous and noncollagenous protein production in groups 4 and 5 at day 10 (but no significance between them). **The most collagenous and noncollagenous protein production was significantly observed in the two-step protocol among the groups at 14 days. *FGF* fibroblast growth factor, *GF* growth factor, *TGF* transforming growth factor

To examine the macromolecules of the ECM, we stained cell cultures with Sirius Red for collagen and Fast Green for noncollagen protein (Fig. 5a, b, c). Looking at gross appearance at 14 days of culture, stains were strongly present in groups 4 and 5, while groups 1 and 3 were weakly stained, and group 2 showed intermediate stain. With a spectrophotometer, the highest collagen and noncollagen protein production was observed in group 5, and the lowest in groups 1 and 3 at 14 days. The most abundant amount of collagen and noncollagen production was significantly observed in group 5 (two-step). At day 10, significantly greater collagen and noncollagen production was also noted in groups 4 and 5, but there was no statistical significance between them.

**Fig. 6:**
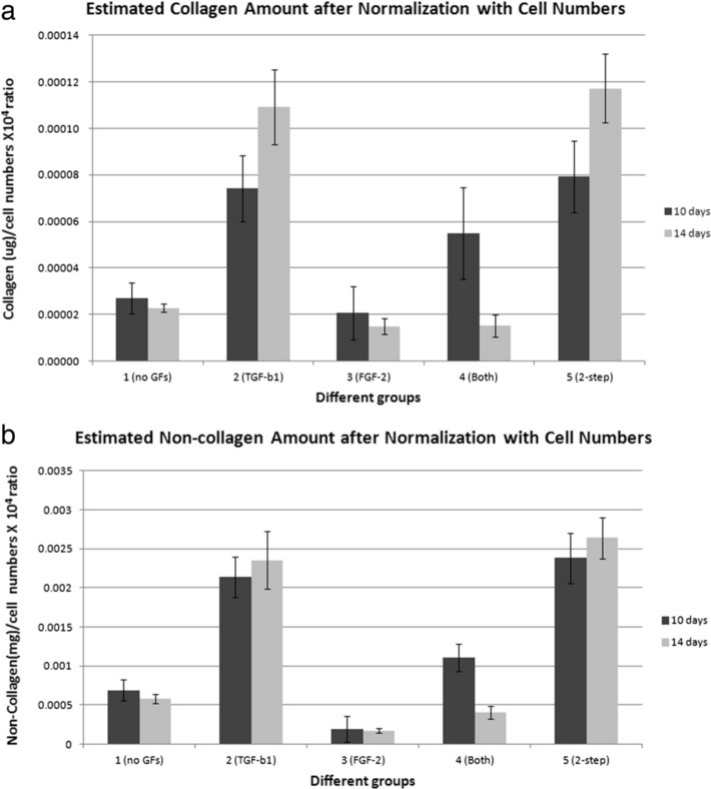
Estimated collagen and noncollagen ECM protein production normalized by cell number (×10^4^) in the five groups at 10 and 14 days of cultivation. The formula for ECM protein calculation was adopted from Gascon-Barre et al. [21]. After normalization for cell number, group 5 (two-step) statistically produced the most collagen and noncollagen ECM, except when compared to group 2 (TGF-β1) (which was statistically insignificant). **a** Estimated collagen amount (μg) normalized for cell number (×10^4^) at 10 and 14 days of cultivation. **b** Estimated noncollagen amount (mg) normalized for cell number (×10^4^) at 10 and 14 days of cultivation. *FGF* fibroblast growth factor, *GF* growth factor, *TGF* transforming growth factor

We adopted the formula from Gascon-Barre et al. [21] to quantify estimated collagen (μg) and noncollagen (mg) ECM proteins. After normalization for cell number, group 5 (two-step) statistically produced the most collagen and noncollagen ECM at 10 and 14 days of cultivation among the five groups, except in comparison to group 2 (which was statistically insignificant; Fig. 6a, b).

**Fig. 7:**
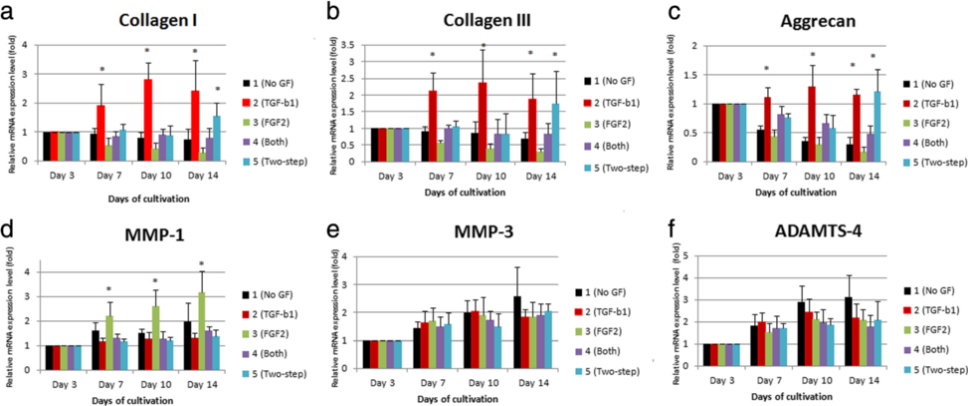
Relative quantitative gene expression in the five groups. Cells (3 × 10^4^) were seeded in P60 dishes for the five groups. Relative quantitative gene expression of (**a**) *collagen I*, (**b**) *collagen III*, (**c**) *aggrecan*, (**d**) *MMP-1*, (**e**) *MMP-3*, and (**f**) *ADAMTS-4* were analyzed at days 3, 7, 10, and 14 by semi-qPCR. Data represent mean ± standard deviation (*n* = 6). Each bar indicates the mean of three experiments in the same group. Statistically high expression of *collagen I*, *collagen III*, and *aggrecan* genes was observed in the TGF-β1 and two-step groups. Statistically lower expression of ECM genes were observed in the FGF-2 group at 7, 10, and 14 days. *Statistically significant upregulated gene expression. *ADAMTS* a disintegrin and metalloproteinase with thrombospondin motif, *FGF* fibroblast growth factor, *GF* growth factor, *MMP* matrix metalloproteinase, *TGF* transforming growth factor

Statistically significant upregulation of *collagen I*, *collagen III*, and *aggrecan* genes was seen in groups 2 and 5 as compared to the other groups at 14 days (Fig. 7). There were trends for upregulation of *collagen I*, *collagen III*, and *aggrecan* gene expression observed in groups 2 and 5 at day 10. Relatively low gene expression of *collagen I*, *collagen III*, and *aggrecan* were observed in group 3 at days 10 and 14. Expression of *MMP-1* was upregulated in the FGF-2 group on different days as compared to the other groups. In addition, the catabolic gene expression of *MMP-3* and *ADAMT-4* were similar between groups at different days of culture.

**Fig. 8:**
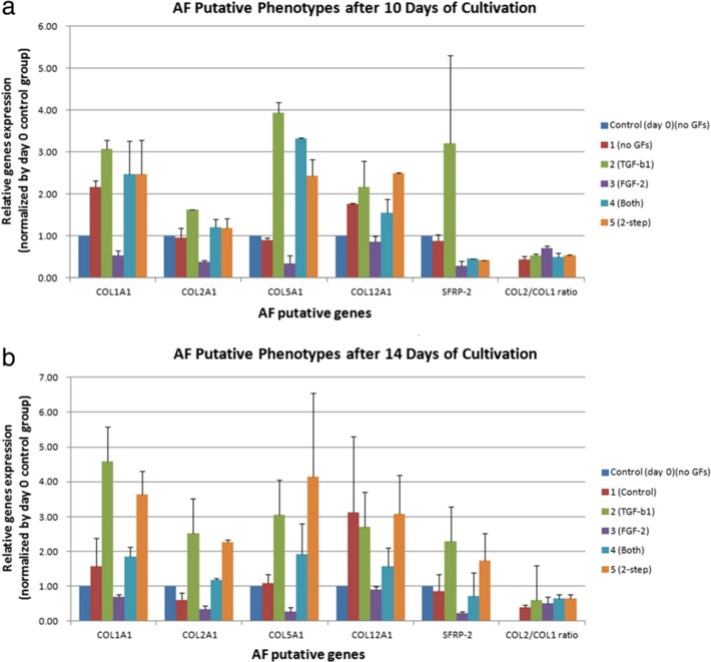
Relative quantitative gene expression of annulus fibrosus (*AF*) putative markers at 10 and 14 days of cultivation in the five groups. The control group did not express the *COL2A1/COL1A1* ratio. The gene expression for *COL1A1*, *COL2A1*, *COL5A1*, *COL12A1*, *SFRP-2*, and *COL2/COL1* ratio were analyzed and compared to those at day 0 of cultivation. Data represent mean ± standard deviation (*n* = 2). AF cells maintained their phenotypes after passage. **a** Relative quantitative gene expression at 10 days of cultivation. **b** Relative quantitative gene expression at 14 days of cultivation. *COL* collagen, *FGF* fibroblast growth factor, *GF* growth factor, *TGF* transforming growth factor, *SFRP* secreted frizzled-related protein

We aimed to confirm the AF phenotype maintenance after passage by measuring expression of putative AF markers. Based on van den Akker et al. [22] and Minogue et al. [23], the characteristics of AF-specific gene expression (Table 1) were relative high expression of *COL1A1*, *COL5A1*, *COL12A1*, and *SFRP2*, and relatively low expression of the *COL2/COL1* ratio as compared to NP cells. Moreover, *SFRP2* was exclusively expressed in primary AF cells [23]. Unfortunately, we did not collect the matched NP tissue for individual disc samples. We compared the selected gene expression at 10 and 14 days of cultivation to those at day 0 (*n* = 2). AF cell phenotypes were only marginally retained under the different protocols based on our qPCR results (Fig. 8).

## Discussion

The goal of the two-step protocol was to synergistically boost cell numbers at the first step by mitogen (FGF-2) and morphogen (TGF-β1), and to obtain more ECM by morphogen (TGF-β1) in the second step during cultivation. Li et al. [7] found that FGF-2 was anti-anabolic and decreased proteoglycan synthesis in bovine disc cells. We also found that FGF-2 had a positive effect on cell numbers (mitogen) but had negative effects on ECM formation in our study. Consequently, we administrated only TGF-β1 in the second step of cultivation to trigger ECM production in conjugation with little anabolic effect on cell proliferation after boosting cells numbers in the first step.

Growth factors (GFs) are fundamental components of the biological homeostasis of ECM in hAF cells [8]. In hAF tissues of degenerated or herniated discs, FGF-2 could be observed by immunohistochemical stain [17, 18]. As a consequence, FGF-2 might participate in the process of disc degeneration or repair in the degenerated stage. We assumed that either the stage of degeneration or the degenerative process could explain the different anabolic or catabolic roles of FGF-2 in the degenerated disc. From our results, FGF-2 could enhance cell proliferation, inhibit ECM production, and downregulate ECM genes relative to collagen I and collagen III in the hAF cells. We may assume that there would be different results if the hAF cells were taken from normal healthy discs postmortem. FGF-2 may create a cascade in hAF tissues, where they act and participate in cellular remodeling from the normal stage to disc degeneration and further herniation.

Previous studies [12, 16, 24] revealed that FGF-2 acted as a mitogen [8] in human disc cells [12] or other species [16, 24]. Pratsinis et al. [12] reported that DNA synthesis in hAF cells obtained from herniated discs was enhanced by PDGF, IGF, and FGF-2 via MEK/ERK and PI-3K/Akt pathways. Thompson et al. [16] reported that FGF-2 enhanced cell proliferation and matrix synthesis in the canine disc. Pratsinis et al. [24] also reported that FGF-2 stimulated cell proliferation of bovine discs via the ERK and Akt signaling pathways. Li et al. [7] showed that FGF-2 had its anti-anabolic effect in a bovine disc via stimulation of MMP-13 production, and inhibition of ECM production via MAPK and NF-kB pathways. We found that FGF-2 could enhance cell proliferation, inhibit ECM production, downregulate ECM genes, and upregulate the MMP-1 gene in our study. The real role of FGF-2 in hAF harvest from degenerated disc tissue still needs to be analyzed to clarify its possible roles in disc degeneration.

Peng et al. [17] and Tolonen et al [18] reported that TGF-β1 was found in human NP (hNP) and posterior AF tissues in the painful degenerated disc via immunochemical staining. No studies have reported the anti-anabolic effect of TGF-β1 on hAF cells. Its appearance in degenerated discs may explain its essential role in repairing or rescuing degeneration. Gruber et al. [10] reported that TGF-β1 (5 ng/mL) enhanced proteoglycan production and cell proliferation for hAF cells in three-dimensional culture. Gruber et al. [13] also reported that TGF-β1 (1 ng/mL) had few mitogenic effects on hAF cells after cultivation for 4 days. We found TGF-β1 (5 ng/mL) had a synergistically mitogenic effect on FGF-2, but much higher morphogenic effects on hAF cells. If we normalized the amount of ECM production for total cell number, we might find each cell in the TGF-β1 group produced more ECM; our goal was also to produce more ECM in the second step, following the boosting of cell production in the first step.

Interleukin (IL)-1 has been identified in the herniated human disc [9, 25]. hNP cells responded to IL-1β via the p38 MAPK signaling pathway [25]. Le Maitre et al. [9] reported that MMP-3, MMP-13, and ADAMTS-4 genes were upregulated following 10 ng/mL IL-1β treatment in degenerated hAF cells. In our study, IL-1β was upregulated not only in the control group, but also in the other groups on different cultivation days. There was a trend in which GF treatment downregulated IL-1β gene expression but did not downregulate other downstream catabolic genes, especially at day 10. Other cytokines besides IL-1β (tumor necrosis factor (TNF)-α, IL-6, IL-8, prostaglandin E2 (PGE2), and nitric oxide (NO) [26, 27]) may also be involved in the complicated pathogenesis of disc degeneration, including upregulation of MMP genes. Monolayer culture, which is different from the naive environment, may accelerate disc degeneration and may explain the gradual upregulation of the IL-1β gene in the control group (data not shown). The results were similar as discovered by Kluba et al [28].

For a clinical application, there are limitations in this study that have to be considered. First, our results were from a monolayer cell culture model, which does not reflect the real three-dimensional disc environment in vivo. Second, there are potentially harmful side effects following locally exogenous GF therapy for AF biologic repair, as reported by Price et al. [29]. Moreover, we did not harvest postmortem healthy disc tissue for our study, which may have less dedifferentiated effects after subsequent cell passage than our protocol. Moreover, we did not quantify each of the corresponding proteins. We might use other GFs in further experiments, which could be more efficient than our two-step protocols. In addition, AF cell phenotypes were only marginally retained under the different protocols. This could be due to the GF effects. In fact, withdrawal of GFs from the cell culture media after 14 days of treatment might shift AF cells back to their original phenotype.

## Conclusion

In summary, the findings in this study have extended the knowledge regarding the effects of FGF-2 and TFG-β1 on hAF cells harvested from degenerated disc tissues. The two-step protocol was more efficient compared to the one-step protocols regarding isolated or combined treatment for hAF cell culture expansion, with moderate cell numbers obtained but most abundant ECM production. The two-step protocol with FGF-2 and TGF-β1 may be a useful formula for hAF culture expansion in future applications for cell therapy of the intervertebral disc.

## Abbreviations

ADMATS-4: a disintegrin and metalloproteinase with thrombospondin motif-4
BrdU: 5-bromo-2’-deoxyuridine
DDD: degenerative disc disease
DMEM: Dulbecco’s modified Eagle medium
ECM: extracellular matrix
FBS: fetal bovine serum
FGF: fibroblast growth factor
GAPDH: glyceraldehyde-3-phosphate dehydrogenase
GF: growth factor
hAF: human annulus fibrosus
hNP: human nucleus pulposus
IGF: insulin-like growth factor
IL: interleukin
MMP: matrix metalloproteinase
NP: nucleus pulposus
PBS: phosphate-buffered saline
PCR: polymerase chain reaction
PDGF: platelet-derived growth factor
TGF: transforming growth factor
